# Texas State Medical Association

**Published:** 1900-03

**Authors:** J. J. Robert, Sam R. Burroughs

**Affiliations:** Chairman; Hillsboro, Texas; Secretary, Buffalo, Texas


### Society Notes.

### TEXAS STATE MEDICAL ASSOCIATION.

### Section on Obstetrics and Diseases of Children.

HILLSBORO, TEXAS, February 1, 1900.

DEAR SIR: We would be glad to have you prepare a paper on some subject for the section on "Obstetrics and Diseases of Children" for the next meeting of the State Medical Association at Waco.

Please forward your paper, or the subject of the same, to the secretary of this section before March 10, if possible.

Respectfully yours,

J. J. ROBERT, Chairman.

SAM R. BURROUGHS, M. D., Secretary, Buffalo, Texas.

